# Reply to L Zhang et al.

**DOI:** 10.1016/j.advnut.2026.100671

**Published:** 2026-06-09

**Authors:** Zhi-Tong Li, Qi-Jun Wu

**Affiliations:** 1 Department of Cardiology, Shengjing Hospital of China Medical University, Shenyang, Liaoning, China; 2 Department of Epidemiology, School of Public Health, China Medical University, Shenyang, Liaoning, China; 3 Key Laboratory of Environment Stress and Chronic Disease Control & Prevention, Ministry of Education, China Medical University, Shenyang, Liaoning, China

Dear Editor:

We sincerely thank Zhang et al. [1] for their thoughtful comments regarding our umbrella review entitled “Prognostic nutritional index and cancer prognostic outcomes: an umbrella review of systematic reviews and meta-analyses of observational studies” [2]. Their letter raises several important considerations concerning the interpretation and clinical applicability of the prognostic nutritional index (PNI) in oncology, which we appreciate as valuable guidance for future research in this field.

We fully agree that PNI is influenced by multiple biological processes beyond nutritional status alone. Serum albumin and lymphocyte count are closely linked to systemic inflammation, tumor burden, immune dysfunction, and cancer-associated cachexia [3,4]. Therefore, low PNI values may indeed reflect underlying disease severity and impaired physiological reserve. In our umbrella review, we did not intend to imply that PNI functions as an isolated or causative prognostic determinant. Rather, our findings support its role as a robust composite biomarker associated with adverse cancer outcomes across diverse malignancies, with 84.5% of the included quantitative syntheses reaching statistical significance under random-effects models [2].

We also acknowledge the important distinction between statistical association and clinical utility. The authors of the letter appropriately highlight that “statistically significant biomarkers do not necessarily improve clinical decision making” [5]. We fully endorse this perspective. As noted in the Reporting recommendations for tumour MARKer prognostic studies (REMARK), the number of markers that have emerged as clinically useful remains pitifully small [6], underscoring the need for rigorous validation beyond initial association studies.

We note that our umbrella review did not make claims regarding the incremental predictive value of PNI beyond conventional clinical variables such as TNM stage, performance status, or treatment method. Instead, the primary objective was to systematically synthesize and critically appraise the breadth, methodological quality, and evidence certainty of existing meta-analyses using A Measurement Tool to Assess Systematic Reviews (AMSTAR) and Grading of Recommendations, Assessment, Development and Evaluation (GRADE) frameworks—an approach that has inherent strengths and limitations [7]. To our knowledge, 64 systematic reviews and meta-analyses comprising 265 quantitative syntheses were included, with 68.8% classified as high quality and 45.7% supported by high-certainty evidence [2]. These findings establish a solid foundation for further investigation into PNI’s incremental prognostic value.

We agree that evidence regarding discrimination, calibration, or reclassification metrics in prognostic models incorporating PNI remains limited. Several recent studies have evaluated PNI-based nomograms with promising results. For example, a PNI-based nomogram for predicting pathological complete response in breast cancer demonstrated satisfactory calibration and discrimination, with C-index values of 0.816 (95% CI: 0.765, 0.866) in the training cohort, 0.780 (95% CI: 0.697, 0.864) in internal validation, and 0.714 (95% CI: 0.660, 0.769) in external validation [8]. A pan-cancer real-world cohort study reported C-indexes for 1-, 3-, 5-, and 10-y overall survival ranging from 0.801 to 0.851 in the validation cohort [9]. These preliminary findings suggest that adding PNI may enhance discrimination beyond traditional staging systems, although further validation across diverse settings is required. Future studies should therefore evaluate whether incorporating PNI into existing prognostic systems meaningfully enhances risk stratification performance using established metrics such as the net reclassification improvement and integrated discrimination improvement.

In addition, we appreciate the discussion regarding the interpretability of PNI as a potentially modifiable target. We agree that observational associations should not be directly interpreted as evidence that interventions aimed at increasing PNI will improve cancer prognosis. Emerging evidence from systematic reviews suggests that nutritional interventions may positively affect quality of life and body weight, but the effect on survival remains less certain [10,11]. As Zhang et al. [1] appropriately note, trials of nutritional supplementation in oncology have shown mixed effects on survival despite improvements in nutritional parameters. Consequently, our review should not be interpreted as supporting causal or intervention-based conclusions. Instead, we concur that PNI may currently be most appropriately regarded as an accessible indicator of host condition, systemic inflammation, and physiological reserve—a marker rather than a target.

Importantly, the clinical relevance of PNI may still extend beyond pure prognostication. Due to its simplicity, low cost, and reproducibility—derived from routine blood tests without additional cost or specialized equipment—PNI may provide practical value in perioperative risk assessment, treatment tolerance evaluation, frailty screening, and identification of patients requiring closer nutritional or supportive management. As Zhang et al. [1] correctly note, however, evidence regarding its actionability remains limited. We fully agree that future research should move beyond demonstrating statistical significance and focus increasingly on clinical integration, incremental prediction, and intervention responsiveness. Their comments provide valuable perspective and help clarify the appropriate interpretation of our findings.

We are grateful to Zhang et al. [1] for emphasizing the conceptual distinction between association, prognostic stratification, and clinical actionability—a distinction that is essential for advancing prognostic research in oncology.

## Declaration of Generative AI and AI-assisted technologies in the writing process

The authors declare that no generative AI or AI-assisted technologies were used in the writing of this manuscript.

## Funding

This work was supported by the Natural Science Foundation of China (No. 82373674 to Q-JW), Liaoning Province Educational Science Planning Project (No. JG22DB707 to Q-JW),Liaoning Province Science and Technology Plan (No. 2023JH2/ 20200019 to Q-JW).

## Conflict of interest

The authors report no conflicts of interest.
